# Supplementary material for: Cortical thickness is differently associated with ALDH2 rs671 polymorphism according to level of amyloid deposition
Source: Sci Rep. 2021 Sep 30;11:19529. doi: 10.1038/s41598-021-98834-8 (PMC8484554; doi:10.1038/s41598-021-98834-8)

Cortical thickness is differently associated with ALDH2 rs671 polymorphism according to level of amyloid deposition

Yong Hyuk Cho^1, 2^, Heirim Lee^3, 4^, Na-Rae Kim^1^, Jin Wook Choi^5^, Hyun Woong Roh^1^, Jae Ho Ha^1^, Chang Hyung Hong^1^, Sang Won Seo^6^, Seong Hye Choi^7^, Eun-Joo Kim^8^, Byeong C. Kim^9^, Seong Yoon Kim^10^, Jaeyoun Cheong^11^, Bumhee Park^3, 4,+^, Sang Joon Son^1,+^

^1^Department of Psychiatry, Ajou University School of Medicine, Suwon, South Korea ^2^ Department of Medical Sciences, Graduate School of Ajou University, Suwon, South Korea ^3^Department of Biomedical Informatics, Ajou University School of Medicine, Suwon, South Korea
^4^Office of Biostatistics, Medical Research Collaborating Center, Ajou Research Institute for innovative Medicine, Ajou University Medical Center, Suwon, South Korea
^5^Department of Radiology, Ajou University School of Medicine, Suwon, South Korea
^6^Department of Neurology, Samsung Medical Center, Sungkyunkwan University School of Medicine, Seoul, South Korea
^7^Department of Neurology, Inha University School of Medicine, Incheon, South Korea
^8^Department of Neurology, Pusan National University School of Medicine, Pusan, South Korea
^9^Department of Neurology, Chonnam National University Medical School, Gwangju, South Korea
^10^Department of Psychiatry, Asan Medical Center, University of Ulsan College of Medicine, Seoul, South Korea
^11^Human Genome Research and Bio-Resource Center, Ajou University Medical Center, Suwon, South Korea

^+^Corresponding authors

**Correspondence:**Sang Joon Son, MD, PhD

Department of Psychiatry, Ajou University School of Medicine

Ajou University School of Medicine, Suwon, Korea 164, World cup-ro, Yeongtong-gu, Suwon-si, Gyeonggi-do, Republic of Korea

Tel: +82-31-219-5180, Fax: +82-31-219-5179, E-mail: sjsonpsy@ajou.ac.kr

Bumhee Park, PhD

Department of Biomedical Informatics, Ajou University School of Medicine

Ajou University School of Medicine, Suwon, Korea 164, World cup-ro, Yeongtong-gu, Suwon-si, Gyeonggi-do, Republic of Korea

Tel: +82-31-219-4458, Fax: +82-31-219-4472, E-mail: bhpark@ajou.ac.kr

**Word count: 2476**

**Number of Tables: 4**

**Number of Figures: 1**

# Abstract

Accumulating evidence indicates that amyloid-beta (Aβ) deposition and biogenic aldehyde accumulation contribute to the pathogenesis of neurodegenerative diseases. Human aldehyde dehydrogenase 2 (ALDH2) metabolizes biogenic aldehydes produced in the brain to prevent damage. However, r671G>A, a single nucleotide polymorphism of ALDH2, causes aldehyde accumulation and decreased ALDH2 activity. We aimed to investigate whether Aβ deposition and rs671 polymorphism have an interaction effect on cortical thickness (CTh). We grouped 179 participants in the Biobank Innovations for chronic Cerebrovascular disease With ALZheimer's disease Study as follows: amyloid (-) [A(-)] and amyloid (+) [A(+)] groups based on the Aβ deposition degree; A-carrier (AC) and GG (GG) groups based on the presence/absence of the rs671 A allele; and their combinations, i.e., A(-)AC, A(-)GG, A(+)AC, and A(+)GG groups. A multiple regression analysis identified nine regions of interest. Compared with the A(-)GG group, the A(-)AC group showed thinner CTh in all regions. There were no significant differences between the A(+)AC and A(+)GG groups. We observed an interaction effect of amyloid deposition and rs671 polymorphism on CTh. The CTh in the A(-) group appeared to be strongly influenced by rs671 polymorphism, which could have contributed to cortical thinning and biogenic aldehyde accumulation in the AC group. Additionally, CTh in the A(+) group appeared to be strongly influenced by amyloid deposition.

Keywords: Aldehyde dehydrogenase 2 (ALDH2), amyloid β (Aβ) deposition, cerebral cortical thickness, neurodegenerative disorder, rs671 polymorphism.

# Introduction

The increasing elderly population has led to a worldwide interest in neurodegenerative disorders, including Alzheimer’s dementia. Numerous studies have reported various causative factors, including environmental and genetic factors, that contribute to the development of neurodegenerative disorders ^1^. Among them, excessive amyloid-β (Aβ) accumulation is an established characteristic of dementia; moreover, most neurodegenerative diseases involve direct and indirect pathologic effects of Aβ. Consequently, there have been various studies on Aβ production, accumulation, and clearance, as well as their interactions with other factors. Recent studies have reported an interaction between Aβ and biogenic aldehydes, including 4-hydroxynonenal (4-HNE) ^2,3^. Aβ-formed fibrils or neuritic plaques have been found to facilitate the production of biogenic aldehydes, which causes brain neurotoxicity; moreover, biogenic aldehydes have been shown to accelerate Aβ-mediated fibril formation ^4,5^. Further, a previous clinical study reported a relationship between amyloid deposition and biogenic aldehydes; it revealed higher hippocampal levels of 4HNE in post-mortem samples obtained from patients with Alzheimer’s disease (AD) ^6^. Additionally, high 4HNE levels have also been reported in hippocampal amyloid plaques and neurofibrillary tangles ^7,8^.

Recent studies on neurodegenerative diseases have investigated human aldehyde dehydrogenase2 (ALDH2), which is crucially involved in the metabolism and detoxification of acetaldehyde generated by ethanol consumption and biogenic aldehydes, including 4-HNE. Moreover, studies have also investigated the rs671 polymorphism, which encodes ALDH2 ^2,3,9^. Specifically, the A allele (ALDH2∗2) of rs671, which is mainly inherent in the East Asian population, intrinsically deregulates ALDH2 activity and induces aldehyde accumulation ^10,11^. Additionally, Chinese and Japanese studies have reported that A-carriers of rs671 (AA/GA) have an increased risk of developing AD ^12,13^.

Based on the aforementioned findings, we aimed to investigate whether amyloid deposition and rs671 polymorphism had interaction effects on brain structural changes. The specific brain structural change we investigated was the cerebral cortex thickness (CTh), which has been used in previous neurodegenerative studies as a diagnostic biomarker, as well as in cognitive function evaluation and prediction ^14-16^. We made the following hypotheses: (1) there is an interaction between amyloid deposition and rs671 polymorphism; (2) under similar amyloid deposition levels, rs671 A-carriers have lower CTh values than GG carriers; and (3) within rs671 A-carriers, amyloid deposition levels are negatively correlated with CTh. The present study aimed to analyze the interaction effects of amyloid deposition and rs671 polymorphism on CTh based on the aforementioned hypotheses.

# Methods

## Participants.

All participants were enrolled in the Biobank Innovations for chronic Cerebrovascular disease With ALZheimer's disease Study (BICWALZS) between November 2016 and December 2019. The BICWALZS was initiated in 2016 by the National Biobank of Korea and Ajou University School of Medicine as a province-wide collaborative biorepository for collecting imaging and genetic resources regarding human biological materials for research in various chronic cerebrovascular diseases. Specifically, this study was designed to identify relationships among individual genetic information, Aβ deposition, and brain structure. We included 251 participants enrolled in the BICWALZS by 2019 with genetic data obtained using DNA chips, standardized uptake value ratio (SUVr) extracted from brain Aβ positron emission tomography (PET), and cerebral CTh data obtained through FreeSurfer after brain magnetic resonance imaging (MRI). Among them, we excluded five patients with frontotemporal dementia who showed severe asymmetrical cerebral cortical atrophy since they did not fit the scope of the study. Moreover, we excluded 67 patients diagnosed with mental illness, which is known to directly affect brain CTh. Finally, we included 179 participants.

The included participants comprised patients with subjective memory impairment, mild cognitive impairment (MCI), Alzheimer's dementia, vascular dementia, dementia with Lewy bodies, and so on. Subjective memory impairment was diagnosed based on self and/or informant report of cognitive decline without impairment in objective cognitive tasks. MCI was diagnosed based on a Clinical Dementia Rating (CDR) ^17^ score of 0.5 and the expanded Mayo Clinic criteria ^18^. Patients with AD were evaluated using the criteria for probable AD following the National Institute on Aging–Alzheimer’s Association core clinical criteria ^19^. Vascular dementia was evaluated using the Diagnostic Statistical Manual, 5^th^ edition (DSM-5) criteria for vascular dementia and above-moderate white matter hyperintensity (WMH). Moreover, dementia with Lewy bodies was evaluated following DSM-5 guidelines. General cognition was evaluated using the Mini Mental Status Examination (MMSE) and dementia severity was measured using the CDR sum of box score.

## Single nucleotide polymorphism (SNP) genotyping and quality control

After informed consent was obtained, all participants provided blood samples for DNA collection and genotyping. The collected blood samples were stored at a biorepository, followed by genomic DNA isolation. SNP genotyping was performed through hybridization on an Affymetrix Axiom KORV1.0-96 Array (Thermo Fisher Scientific, Waltham, MA) following the manufacturer’s instructions. Genotype data were produced using K-CHIPs designed by the Center for Genome Science, Korea National Institute of Health, Republic of Korea (4845–301, 3000–3031). Genotyping was performed using DNA Link (Seoul, Republic of Korea). The ALDH2 genotype was derived from rs671, which was included in the genotyping array. Given that rs671G>A significantly reduces ALDH2 activity, we divided the participants into the AC group (ALDH2*1/*2 and ALDH2*2/*2) and GG group (ALDH*1/*1) based on the presence of ALDH2*2 in rs671.

Quality control was performed using PLINK (version 1.07; Free Software Foundation, Boston, MA, USA). We excluded samples with an individual call-rate < 97%, inconsistency between the reported sex and X-chromosome SNP analysis, and extremely low or high genome-wide heterozygosity (±2 standard deviation [SD] from the mean). Moreover, we excluded samples with SNPs that had a call-rate < 95% and a Hardy-Weinberg equilibrium test p-value < 10^-6^.

## MRI acquisition and measurement of CTh and WMH

MRI data were obtained using a 3.0 Tesla MR scanner. We obtained structural MRI scans, including 3D T1, T2, and fluid-attenuated inversion recovery (FLAIR) scans. All MR images were reviewed by neuroradiologists. Detailed MRI parameters according to the site have been described in **Supplementary Table S1**. All T1-weighted images were preprocessed with FreeSurfer (http://surfer.nmr.mgh.harvard.edu/, version 6.0) using the “recon-all” pipeline ^20,21^. This procedure involved automatic surface-based processing, including affine registration to the Talairach atlas, intensity normalization, bias correction, and skull stripping ^22-25^. Additionally, the processing steps involved the segmentation of anatomical brain images into the gray matter (pial matter), white matter, and subcortical structures, with correction of each tissue/structure boundary. Further, T2 FLAIR images were input on the “recon-all” command line to improve between-matter contrast differentiation. Surface segmentations were visually checked, and surface misplacement errors were manually fixed by editing the “brainmask.mgz” image, which is used for skull stripping, by a well-trained researcher or physician. Subsequently, the edited images underwent further surface extraction. CTh was calculated as the mean distance between the gray and white matter surfaces. We extracted the mean CTh of 68 brain regions based on the Desikan-Killiany atlas ^26^ using the “mri_segstats” command ^27,28^.

WMH quantification was performed using a semi-automatic segmentation method in the nordicICE Basis Module ^29^. Specifically, on the FLAIR images, WMH was defined as white matter pixel values > 2 SD above the mean pixel value of each slice. The total WMH area in all the slices was summed and multiplied with the slice thickness to yield the total WMH volume (mL). WMH was separately examined in periventricular and deep white matter lesions.

## Brain amyloid PET acquisition

All participants underwent an ^18^F-flutemetamol PET scan to quantify the cortical Aβ burden using a Discovery Ste (GE), Biograph40 (SIEMENS), or Discovery 690(GE) PET/CT scanner. A bolus of ^18^F-flutemetamol was injected into an antecubital vein with a mean dose of 185 or 188.7 MBq. After 90 min, a 20-min PET scan was performed. The resulting ^18^F-flutemetamol PET scans were co-registered with individual MRI scans, which were normalized to a T1-weighted MRI template. Using transformation parameters, we normalized the MRI-co-registered ^18^F-flutemetamol PET images to the MRI template. To quantify ^18^F-flutemetamol retention, the SUVr was obtained with the pons as a reference region. We calculated the global cortical ^18^F-flutemetamol retention from the volume-weighted mean SUVr of 28 bilateral cortical volumes of interest from the frontal, posterior cingulate, lateral temporal, parietal, and occipital lobes using the Annotated Anatomical Labelling atlas ^30^. We speculated that the interaction effects between Aβ deposition and rs671 polymorphism differed depending on brain Aβ deposition levels. Therefore, we performed comparisons based on the Aβ deposition level. Specifically, based on a previous report on the elderly Korean population and our observed data distribution, we divided participants into the Aβ (+) and Aβ (-) groups if their global cortical SUVr was > 0.65 and < 0.65, respectively ^31^.

## Cognitive function assessment

Cognitive function was evaluated using the Seoul Neuropsychological Screening Battery, which comprises tests on language, visuospatial, memory, and frontal/executive function. General cognition and dementia severity were evaluated using the Korean-MMSE (K-MMSE) and CDR sum of box, respectively. Attention, language, and visuospatial function were evaluated using the digit span backward, Boston Naming Test (range: 0–60), and Rey Complex Figure Test (range: 0–36), respectively. Memory function was calculated by summing the scores of verbal (Seoul Verbal Learning Test immediate recall, delayed recall, and recognition score) and visual memory tests (Rey Complex Figure Test immediate recall, delayed recall, and recognition score; range: 0–144). Frontal/executive function was evaluated by summing the scores of the Controlled Oral Word Association Test and Stroop Color Reading Test (range: 0–55).

## Consideration of potential confounding variables

In the multiple linear regression analysis, we considered several confounding variables that could affect the change of brain cortical thickness and ALDH2 rs671 polymorphism, as shown in the previous studies^32^. First, we adjusted the effects of age, sex, total intracranial volume (TIV), APOE-ε4 carrier status, and education years since the effects can basically affect cortical thickness. CDR sum of box was also considered since the severity of neurodegenerative diseases can influence cortical thickness^33,34^. Since rs671 is involved in the metabolism of aldehydes produced by alcohol consumption, the lifetime drinking status was included as a covariate^35^. The lifetime drinking status was classified into “Drinker” and “Non-drinker,” where “Drinkers” and “Non-drinkers” were defined as those who had ever and never consumed alcohol, respectively. In addition, many recent studies have reported the association of rs671 with cardiovascular disorders and cerebrovascular diseases, and these diseases are known to affect the thickness of the cerebral cortex^36^. For this reason, we tried to comprehensively correct the effects of cardiovascular disease by selecting the Framingham General Cardiovascular Risk Score (FGCRS), which has been internationally used in recent studies on neurodegenerative diseases as a covariate to adjust the cardiovascular risk as a variable^37^ as well as hypertension, dyslipidemia, and diabetes mellitus. The FGCRS(%) is calculated as the sum of weighted measures of age, sex, systolic blood pressure, high-density lipoprotein levels, total cholesterol levels, smoking status, and diabetes status, which was subsequently converted to a percentage. The scores are positively correlated with cardiovascular risk^38^. Finally, we tried to correct the effect on the cerebrovascular disease by selecting the WMH score of the peripheral and deep white matter^39,40^.

**Group categorization and statistical analysis**

The included participants were divided into the following groups: the amyloid (-)[A(-)] and amyloid (+) groups [(A(+)] based on the Aβ deposition degree; the A-carrier (AC) and GG (GG) groups based on the presence/absence of rs671 A allele; and the combination of the aforementioned groups: A(-)AC, A(-)GG, A(+)AC, and A(+)GG groups. Demographic characteristics were compared between AC and GG within each amyloid group using the t-test, chi-squared test. Also, in this study, we compared continuous demographic variables, clinical variables, and cognitive function scores among the four groups (i.e., A(-)AC, A(-)GG, A(+)AC, and A(+)GG) using ANOVA and performed post-hoc tests once each result from ANOVA showed significant difference. Post-hoc tests were adjusted with Bonferroni correction (P<0.05).

To determine the interaction effects of Aβ deposition and rs671 polymorphism on CTh, we performed multiple regression analysis with false discovery rate (FDR) correction. Statistical significance for multiple comparisons was set at an FDR threshold of < 0.05. FDR thresholding controls the expected false-positive proportion to only brain regions showing significance. After identifying a specific brain region through multiple regression analysis, we performed between-group comparisons to determine significant differences in the CTh of each brain region, which were reported as the least-square means (Lsmeans) and p-values. We conducted Bonferroni’s correction for multiple comparison error. Statistical significance was set at a corrected p-value < 0.05.

Analysis was conducted in R software (version 3.5.3; R Core Team [2014]; R: A language and environment for statistical computing; R Foundation for Statistical Computing, Vienna Austria; http://www.r-project.org/). In the Figure 1, visualization of brain maps was performed using R-package ggseg (https://github.com/ggseg/ggseg)^41^ and bar plots were drawn using the R-package ggplot2^42^.

## Ethical approval

The BICWALZS was registered at the Clinical Research Information Service (CRiS) of Republic of Korea(identifier: KCT0003391) and approved by the Institutional Review Board of Ajou University Hospital (AJIRB-BMR-SUR-16-362). It complied with the 1964 Helsinki declaration and its later amendments or comparable ethical standards. Written informed consent was obtained from all participants and caregivers.

# Results

## Sample characterization

**Table 1** summarizes the demographic characteristics of the included participants grouped according to the SUVr on Aβ PET and the presence of rs671 A allele. The A(-) group comprised 87 (48.6%) participants; among them, 62 and 25 participants were in the GG and AC groups, respectively, with MCI being the most common diagnosis (7 [68.0%], A(-)AC; 35 [56.5%], A(-)GG). The A(+) group comprised 92 (51.4%) participants; among them, 31 and 61 participants were in the AC and GG groups, respectively, with Alzheimer's dementia being the most common diagnosis (18 [58.1%], A(+)AC; 32 (52.5%) A(+)GG). In both amyloid groups, there were no significant differences in K-MMSE scores, CDR sum of boxes scores, hypertension, diabetes mellitus, and dyslipidemia between AC and GG. However, the FGCRS(%) was significantly higher in the A(-)AC group (16.560%) than in the A(-)GG group (14.194). Regarding lifetime alcohol drinking, there was a significantly higher ratio of non-drinkers in the AC group than in the GG group for both amyloid groups, with this difference being significant in the A(+) group [A(+)AC, 87.1%; A(+)GG, 59.0%]. This is consistent with previous reports of a low incidence of alcohol use disorder and alcohol consumption in patients with rs671G>A ^35^.

Regarding age, the A(-)GG group was significantly younger than the A(+)GG group (70.774 years vs. 74.508 years). The A(-)AC group had significantly lower SUVr values than the A(+)AC group (0.576 vs. 0.865); moreover, the A(-)GG group had significantly lower SUVr values than the A(+)GG group (0.579 vs. 0.913). Within the same genotype, there were significant differences in APOE- ε4 status and cognitive diagnosis between the amyloid groups. **Supplementary Table S2, S3** presents the between-group comparisons of the demographic characteristics.

## Cognitive function

The ANOVA test results for among 4 groups(i.e., A(-)AC, A(-)GG, A(+)AC, and A(+)GG) are presented in **Table 2**. In the comparison among 4 groups, there were differences in visuospatial function, memory function and frontal/executive function. In addition, as a result of the post-hoc test, the memory function was statistically significantly decreased in the A(+)AC and A(+)GG groups compared to the A(-)AC and A(-)GG groups, respectively.

## **The interaction effect between Aβ deposition and rs671 polymorphism on brain cortical thickness**

To determine the interaction effects between Aβ deposition and rs671 polymorphism on brain CTh, we performed multiple regression analyses with FDR correction. As shown in **Table 3**, we identified nine areas with FDR corrected *p*-values < 0.05. The analysis results for a total of 68 areas divided into Desikan-Killiany atlas are displayed in **Supplementary Table S4**.

Subsequently, we performed among-group comparisons of the CTh in the nine regions. **Table 4** presents the Lsmeans and their respective p-values, which are visualized in **Figure 1**. A similar pattern was observed in all nine regions; moreover, the A(-)AC group showed significantly lower CTh values in all regions than the A(-)GG groups at a Bonferroni’s corrected *p*-value < 0.05. Contrastingly, there were no significant differences between the A(+)AC and A(+)GG groups. Compared with the A(-)GG and A(+)GG groups, the A(+)GG group showed lower Lsmeans of CTh in eight of the nine areas; among them, the differences were significant in the Lt. superior parietal, Rt. inferior parietal, and Rt. superior parietal. In contrast to our expected finding that the A(+)AC group had the lowest Lsmeans of CTh, this was observed in the A(-)AC group. Moreover, compared with the A(-)AC group, the A(+)AC group showed significantly higher CTh in the Lt. cuneus, Lt. pars opercularis, Lt. pars triangularis, and Lt. insula.

# DISCUSSION

Our findings suggest that amyloid deposition and rs671 polymorphism have interaction effects on CTh in older patients. These findings are important since they were obtained after adjustment for various clinical factors, including the vascular load, which affects CTh. Specifically, we observed a genotype-based (presence/absence of rs671G>A polymorphism) influence on the amyloid deposition effect on CTh among individuals in East Asia, including the Republic of Korea. Therefore, future studies on brain volume reduction resulting from amyloid deposition in older patients should consider their genetic characteristics.

First of all, we observed interesting results compared between the A(-) and the A(+) groups within the same rs671 genotype. Participants in the A(-)GG and A(+)GG groups who did not show a functional decline in aldehyde processing, revealed cortical thinning in the bilateral superior parietal and Rt. inferior parietal. This is consistent with previous reports regarding the areas where initial cortical thinning occurs due to amyloid deposition ^43^. However, we observed notable findings after comparing amyloid deposition effects within the AC group. Specifically, there were higher CTh values in the A(+)AC group than in the A(-)AC group in all nine regions, with four regions showing significant differences. However, the A(+)AC group showed lower scores for all cognitive domains, which was significant for memory function. This is indicative of differences between the effects on CTh and cognitive function scores. Even if the CTh values contradicted the hypothesis, our findings can be explained with the biphasic change trajectory for CTh reported in recent animal ^44,45^ and human studies ^46,47^. The biphasic trajectory in cross-sectional ^46,48^ and longitudinal studies ^49^ suggests that pathological cortical thickening occurs during the initial period of AD progression, which is more pronounced in the temporal lobe than in the frontal lobe ^50,51^. In biological terms, amyloid-related CTh can be described as an inflammatory response to oligomeric Aβ, Aβ-induced neuronal hypertrophy, pathogenic synergies between Aβ and Tau, and relationships between other factors ^52,53^. These findings suggest that the effect of amyloid deposition on CTh varies depending on rs671 polymorphism. Several recent studies on CTh have suggested that synergistic effects of amyloid and tau, rather than amyloid deposition alone, are crucially involved in cortical thinning; moreover, atrophy in the early disease stages is associated with these synergistic effects ^46,54^. However, tau pathology primarily occurs in the late AD stage; on the other hand, neuroinflammation and astrocyte activation are major pathological occurrences in the early AD stages ^55^. Specifically, numerous studies have shown that inflammation causes neuronal and glial swelling, as well as glia recruitment and activation, which presents increased CTh in the early AD stages ^47,56^. Further, biogenic aldehydes are strongly associated with oxidative stress and inflammation; moreover, they are known to accelerate Aβ-mediated fibril formulation ^4,5^. Taken together, these findings indicate that although cognitive decline was relatively more advanced in the A(+)AC group, there were alternative interactions other than the aforementioned interaction between amyloid deposition and rs671 polymorphism in the A(-) group. This suggests that excessive inflammation related to abnormalities in biogenic aldehyde metabolism observed in the AC group may result in increased paradoxical cortical thickening during early-stage degenerative changes caused by cell swelling, glial cell recruit/activation, and Aβ fibril formation. Moreover, these phenomena have been mainly reported in the insular and inferior frontal regions rather than the areas presenting initial cortical thinning due to amyloid deposition.

Subsequently, a comparison between AC and GG within the same amyloid group observed different results depending on the amyloid group. There was no significant difference in CTh between the A(+)AC and A(+)GG groups in all nine areas. Contrastingly, the A(-)AC group showed significantly lower CTh values than the A (-)GG group in all nine regions (**Figure 1, Table 4**). The A(-) group comprised a heterogeneous population mainly diagnosed with MCI and subjective memory impairment. Notably, the global SUVr value in the A(-)AC and A(-)GG groups were 0.576 and 0.579, respectively, which is similar to the value of 0.58 reported by a previous study on elderly Korean individuals with normal cognitive function^31^. Our results suggest that in normal individuals with amyloid deposition, differences in rs671 polymorphism significantly affect the CTh. This can be interpreted by the relationship between neurodegenerative changes and biogenic aldehydes, which has recently accumulated extensive evidence ^6,8,57,58^. Biogenic aldehydes generated in the brain are metabolized by ALDH2 expressed in neurons and glial cells present in the frontal lobe, temporal lobe, hippocampus, midbrain, basal ganglia, cerebellum, etc. This process facilitates the minimization of aldehyde-induced brain damage^59^. However, ALDH2 activity is significantly reduced if rs671, which is known as the SNP of ALDH2, is AG and AA; specifically, rs671G>A is associated with an increased incidence of neurodegenerative diseases ^60^. Our findings suggest that compared with the A(-)GG group, the A(-)AC group showed lower CTh values, which resulted from brain damage caused by abnormalities in the processing of biogenic aldehydes. The nine identified areas were mainly frontoparietal; moreover, previous studies have reported various areas with cortical thinning associated with aging, dementia with Lewy bodies, or other mental disorders ^16,61,62^. This could be attributed to the heterogeneity in the A(-) group. We corrected FGCRS and WMH with reference to a recent study that reported a relationship between vascular load and lipid profile in relation to rs671 ^10,39,40,63,64^. Despite performing these corrections, we assumed that several factors, including other vascular factors not addressed in this study, may have affected the findings.

This study has several limitations. First, this was a small-scale cross-sectional study. Additionally, the study population was heterogeneous, especially in the A(-) group, and comprised elderly individuals with various clinical diagnoses and cognitive symptoms. Second, since we employed the global SUVr, we could not determine the correlation between SUVr and the CTh of each brain region. Third, other than FGCRS(%) and WMH, we did not analyze tau protein, inflammatory factors, and other vascular factors that could directly affect brain blood flow, including vessel tortuosity, lipohyalinosis, arteriolosclerosis, venous collagenosis, blood-brain barrier disruption, and vessel obliteration ^65^. Finally, this study is a candidate gene study conducted by selecting rs671. A candidate gene study has been criticized for its gene selection process and nonreplication of results. In addition, it has a limitation in that it shows a high number of false positives because all genes that contribute to the cause cannot be considered^66^.

In summary, we observed interaction effects of amyloid deposition and rs671 polymorphism on CTh. The CTh in the A(-) group appeared to be heavily influenced by rs671 polymorphism, which could have contributed to cortical thinning and biogenic aldehyde accumulation in the AC group. Additionally, CTh in the A(+) group appeared to be heavily influenced by amyloid deposition. The paradoxical phenomenon of decreased and increased cognitive function and CTh, respectively, in the AC group suggests that the interaction between amyloid deposition and a certain inflammatory response level in the AC group, which presented reduced ALDH2 activity, increases the CTh during the initial stage of degenerative changes though cell swelling, as well as glial cell recruitment and activation. Future studies on brain volume reduction related to amyloid deposition in older patients should consider their genetic characteristics. In addition, research focusing on a number of factors that can affect an individual's CTh, such as inflammation and tau pathology, as well as amyloid deposition and aldehyde accumulation, needs to be conducted to clarify the relationship between these factors.

# REFERENCES

1 Querfurth, H. W. & LaFerla, F. M. Alzheimer's disease. *N Engl J Med* **362**, 329-344, doi:10.1056/NEJMra0909142 (2010).

2 Chen, C. H., Joshi, A. U. & Mochly-Rosen, D. The Role of Mitochondrial Aldehyde Dehydrogenase 2 (ALDH2) in Neuropathology and Neurodegeneration. *Acta Neurol Taiwan* **25(4)**, 111-123 (2016).

3 Joshi, A. U. *et al.* Aldehyde dehydrogenase 2 activity and aldehydic load contribute to neuroinflammation and Alzheimer's disease related pathology. *Acta Neuropathol Commun* **7**, 190, doi:10.1186/s40478-019-0839-7 (2019).

4 Koppaka, V. & Axelsen, P. H. Accelerated accumulation of amyloid beta proteins on oxidatively damaged lipid membranes. *Biochemistry* **39**, 10011-10016, doi:10.1021/bi000619d (2000).

5 Koppaka, V., Paul, C., Murray, I. V. & Axelsen, P. H. Early synergy between Abeta42 and oxidatively damaged membranes in promoting amyloid fibril formation by Abeta40. *J Biol Chem* **278**, 36277-36284, doi:10.1074/jbc.M301334200 (2003).

6 Williams, T. I., Lynn, B. C., Markesbery, W. R. & Lovell, M. A. Increased levels of 4-hydroxynonenal and acrolein, neurotoxic markers of lipid peroxidation, in the brain in Mild Cognitive Impairment and early Alzheimer's disease. *Neurobiol Aging* **27**, 1094-1099, doi:10.1016/j.neurobiolaging.2005.06.004 (2006).

7 Ando, Y. *et al.* Histochemical detection of 4-hydroxynonenal protein in Alzheimer amyloid. *J Neurol Sci* **156**, 172-176, doi:10.1016/s0022-510x(98)00042-2 (1998).

8 Sayre, L. M. *et al.* 4-Hydroxynonenal-derived advanced lipid peroxidation end products are increased in Alzheimer's disease. *J Neurochem* **68**, 2092-2097, doi:10.1046/j.1471-4159.1997.68052092.x (1997).

9 Cagle, B. S., Crawford, R. A. & Doorn, J. A. Biogenic Aldehyde-Mediated Mechanisms of Toxicity in Neurodegenerative Disease. *Curr Opin Toxicol* **13**, 16-21, doi:10.1016/j.cotox.2018.12.002 (2019).

10 Chen, C. H. *et al.* Activation of aldehyde dehydrogenase-2 reduces ischemic damage to the heart. *Science* **321**, 1493-1495, doi:10.1126/science.1158554 (2008).

11 Lai, C. L. *et al.* Dominance of the inactive Asian variant over activity and protein contents of mitochondrial aldehyde dehydrogenase 2 in human liver. *Alcohol Clin Exp Res* **38**, 44-50, doi:10.1111/acer.12215 (2014).

12 Kamino, K. *et al.* Deficiency in mitochondrial aldehyde dehydrogenase increases the risk for late-onset Alzheimer's disease in the Japanese population. *Biochem Biophys Res Commun* **273**, 192-196, doi:10.1006/bbrc.2000.2923 (2000).

13 Ma, L. & Lu, Z. N. Role of ADH1B rs1229984 and ALDH2 rs671 gene polymorphisms in the development of Alzheimer's disease. *Genet Mol Res* **15**, doi:10.4238/gmr.15048740 (2016).

14 Hwang, J. *et al.* Prediction of Alzheimer's disease pathophysiology based on cortical thickness patterns. *Alzheimers Dement (Amst)* **2**, 58-67, doi:10.1016/j.dadm.2015.11.008 (2016).

15 Racine, A. M., Brickhouse, M., Wolk, D. A., Dickerson, B. C. & Alzheimer's Disease Neuroimaging, I. The personalized Alzheimer's disease cortical thickness index predicts likely pathology and clinical progression in mild cognitive impairment. *Alzheimers Dement (Amst)* **10**, 301-310, doi:10.1016/j.dadm.2018.02.007 (2018).

16 Zarei, M. *et al.* Cortical thinning is associated with disease stages and dementia in Parkinson's disease. *J Neurol Neurosurg Psychiatry* **84**, 875-881, doi:10.1136/jnnp-2012-304126 (2013).

17 Morris, J. C. The Clinical Dementia Rating (CDR): current version and scoring rules. *Neurology* **43**, 2412-2414, doi:10.1212/wnl.43.11.2412-a (1993).

18 Winblad, B. *et al.* Mild cognitive impairment--beyond controversies, towards a consensus: report of the International Working Group on Mild Cognitive Impairment. *J Intern Med* **256**, 240-246, doi:10.1111/j.1365-2796.2004.01380.x (2004).

19 McKhann, G. M. *et al.* The diagnosis of dementia due to Alzheimer's disease: recommendations from the National Institute on Aging-Alzheimer's Association workgroups on diagnostic guidelines for Alzheimer's disease. *Alzheimers Dement* **7**, 263-269, doi:10.1016/j.jalz.2011.03.005 (2011).

20 Dale, A. M., Fischl, B. & Sereno, M. I. Cortical surface-based analysis. I. Segmentation and surface reconstruction. *Neuroimage* **9**, 179-194, doi:10.1006/nimg.1998.0395 (1999).

21 Fischl, B., Sereno, M. I. & Dale, A. M. Cortical surface-based analysis. II: Inflation, flattening, and a surface-based coordinate system. *Neuroimage* **9**, 195-207, doi:10.1006/nimg.1998.0396 (1999).

22 Fischl, B. *et al.* Whole brain segmentation: automated labeling of neuroanatomical structures in the human brain. *Neuron* **33**, 341-355, doi:10.1016/s0896-6273(02)00569-x (2002).

23 Fischl, B. *et al.* Sequence-independent segmentation of magnetic resonance images. *Neuroimage* **23 Suppl 1**, S69-84, doi:10.1016/j.neuroimage.2004.07.016 (2004).

24 Segonne, F., Pacheco, J. & Fischl, B. Geometrically accurate topology-correction of cortical surfaces using nonseparating loops. *IEEE Trans Med Imaging* **26**, 518-529, doi:10.1109/TMI.2006.887364 (2007).

25 Sled, J. G., Zijdenbos, A. P. & Evans, A. C. A nonparametric method for automatic correction of intensity nonuniformity in MRI data. *IEEE Trans Med Imaging* **17**, 87-97, doi:10.1109/42.668698 (1998).

26 Desikan, R. S. *et al.* An automated labeling system for subdividing the human cerebral cortex on MRI scans into gyral based regions of interest. *Neuroimage* **31**, 968-980, doi:10.1016/j.neuroimage.2006.01.021 (2006).

27 Destrieux, C., Fischl, B., Dale, A. & Halgren, E. Automatic parcellation of human cortical gyri and sulci using standard anatomical nomenclature. *Neuroimage* **53**, 1-15, doi:10.1016/j.neuroimage.2010.06.010 (2010).

28 Fischl, B. *et al.* Automatically parcellating the human cerebral cortex. *Cereb Cortex* **14**, 11-22, doi:10.1093/cercor/bhg087 (2004).

29 Li, Y. *et al.* Compromised Blood-Brain Barrier Integrity Is Associated With Total Magnetic Resonance Imaging Burden of Cerebral Small Vessel Disease. *Front Neurol* **9**, 221, doi:10.3389/fneur.2018.00221 (2018).

30 Tzourio-Mazoyer, N. *et al.* Automated anatomical labeling of activations in SPM using a macroscopic anatomical parcellation of the MNI MRI single-subject brain. *Neuroimage* **15**, 273-289, doi:10.1006/nimg.2001.0978 (2002).

31 Hwang, J. *et al.* Clinical and Biomarker Characteristics According to Clinical Spectrum of Alzheimer's Disease (AD) in the Validation Cohort of Korean Brain Aging Study for the Early Diagnosis and Prediction of AD. *J Clin Med* **8**, doi:10.3390/jcm8030341 (2019).

32 Parker, T. D. *et al.* Amyloid β influences the relationship between cortical thickness and vascular load. *Alzheimers Dement (Amst)* **12**, e12022, doi:10.1002/dad2.12022 (2020).

33 Kim, S. E. *et al.* Cognitive trajectories of patients with focal ss-amyloid deposition. *Alzheimers Res Ther* **13**, 48, doi:10.1186/s13195-021-00787-7 (2021).

34 Kim, K. W. *et al.* Disease progression modeling of Alzheimer's disease according to education level. *Sci Rep* **10**, 16808, doi:10.1038/s41598-020-73911-6 (2020).

35 Luczak, S. E., Glatt, S. J. & Wall, T. L. Meta-analyses of ALDH2 and ADH1B with alcohol dependence in Asians. *Psychol Bull* **132**, 607-621, doi:10.1037/0033-2909.132.4.607 (2006).

36 Zheng, Y. *et al.* Association Between ALDH-2 rs671 and Essential Hypertension Risk or Blood Pressure Levels: A Systematic Review and Meta-Analysis. *Frontiers in Genetics* **11**, doi:10.3389/fgene.2020.00685 (2020).

37 Song, R. *et al.* Associations Between Cardiovascular Risk, Structural Brain Changes, and Cognitive Decline. *J Am Coll Cardiol* **75**, 2525-2534, doi:10.1016/j.jacc.2020.03.053 (2020).

38 D'Agostino, R. B., Sr. *et al.* General cardiovascular risk profile for use in primary care: the Framingham Heart Study. *Circulation* **117**, 743-753, doi:10.1161/CIRCULATIONAHA.107.699579 (2008).

39 Huang, Y. H., Chang, K. H., Lee, Y. S., Chen, C. M. & Chen, Y. C. Association of alcohol dehydrogenase and aldehyde dehydrogenase Polymorphism with Spontaneous Deep Intracerebral Haemorrhage in the Taiwan population. *Sci Rep* **10**, 3641, doi:10.1038/s41598-020-60567-5 (2020).

40 Zhu, Z. *et al.* ALDH2 rs671 polymorphisms and the risk of cerebral microbleeds in Chinese elderly: the Taizhou Imaging Study. *Ann Transl Med* **8**, 229, doi:10.21037/atm.2020.01.01 (2020).

41 Mowinckel, A. M. & Vidal-Piñeiro, D. Visualization of Brain Statistics With R Packages ggseg and ggseg3d. *Advances in Methods and Practices in Psychological Science* **3**, 466-483, doi:10.1177/2515245920928009 (2020).

42 Wickham, H. *ggplot2: Elegant Graphics for Data Analysis*. (Springer-Verlag New York, 2016).

43 Becker, J. A. *et al.* Amyloid-beta associated cortical thinning in clinically normal elderly. *Ann Neurol* **69**, 1032-1042, doi:10.1002/ana.22333 (2011).

44 Grand'maison, M. *et al.* Early cortical thickness changes predict beta-amyloid deposition in a mouse model of Alzheimer's disease. *Neurobiol Dis* **54**, 59-67, doi:10.1016/j.nbd.2013.02.005 (2013).

45 Hebert, F. *et al.* Cortical atrophy and hypoperfusion in a transgenic mouse model of Alzheimer's disease. *Neurobiol Aging* **34**, 1644-1652, doi:10.1016/j.neurobiolaging.2012.11.022 (2013).

46 Fortea, J. *et al.* Cerebrospinal fluid beta-amyloid and phospho-tau biomarker interactions affecting brain structure in preclinical Alzheimer disease. *Ann Neurol* **76**, 223-230, doi:10.1002/ana.24186 (2014).

47 Montal, V. *et al.* Cortical microstructural changes along the Alzheimer's disease continuum. *Alzheimers Dement* **14**, 340-351, doi:10.1016/j.jalz.2017.09.013 (2018).

48 Quiroz, Y. T. *et al.* Brain Imaging and Blood Biomarker Abnormalities in Children With Autosomal Dominant Alzheimer Disease: A Cross-Sectional Study. *JAMA Neurol* **72**, 912-919, doi:10.1001/jamaneurol.2015.1099 (2015).

49 Pegueroles, J. *et al.* Longitudinal brain structural changes in preclinical Alzheimer's disease. *Alzheimers Dement* **13**, 499-509, doi:10.1016/j.jalz.2016.08.010 (2017).

50 La Joie, R. *et al.* Region-specific hierarchy between atrophy, hypometabolism, and beta-amyloid (Abeta) load in Alzheimer's disease dementia. *J Neurosci* **32**, 16265-16273, doi:10.1523/JNEUROSCI.2170-12.2012 (2012).

51 Bakkour, A., Morris, J. C., Wolk, D. A. & Dickerson, B. C. The effects of aging and Alzheimer's disease on cerebral cortical anatomy: specificity and differential relationships with cognition. *Neuroimage* **76**, 332-344, doi:10.1016/j.neuroimage.2013.02.059 (2013).

52 Dickerson, B. C. *et al.* The cortical signature of Alzheimer's disease: regionally specific cortical thinning relates to symptom severity in very mild to mild AD dementia and is detectable in asymptomatic amyloid-positive individuals. *Cereb Cortex* **19**, 497-510, doi:10.1093/cercor/bhn113 (2009).

53 Johnson, S. C. *et al.* Amyloid burden and neural function in people at risk for Alzheimer's Disease. *Neurobiol Aging* **35**, 576-584, doi:10.1016/j.neurobiolaging.2013.09.028 (2014).

54 Kobe, T. *et al.* Association of Vascular Risk Factors With beta-Amyloid Peptide and Tau Burdens in Cognitively Unimpaired Individuals and Its Interaction With Vascular Medication Use. *JAMA Netw Open* **3**, e1920780, doi:10.1001/jamanetworkopen.2019.20780 (2020).

55 Rodriguez-Vieitez, E. *et al.* Diverging longitudinal changes in astrocytosis and amyloid PET in autosomal dominant Alzheimer's disease. *Brain* **139**, 922-936, doi:10.1093/brain/awv404 (2016).

56 Vilaplana, E. *et al.* Cortical microstructural correlates of astrocytosis in autosomal-dominant Alzheimer disease. *Neurology* **94**, e2026-e2036, doi:10.1212/WNL.0000000000009405 (2020).

57 Butterfield, D. A., Castegna, A., Lauderback, C. M. & Drake, J. Evidence that amyloid beta-peptide-induced lipid peroxidation and its sequelae in Alzheimer's disease brain contribute to neuronal death. *Neurobiol Aging* **23**, 655-664, doi:10.1016/s0197-4580(01)00340-2 (2002).

58 Furman, R. *et al.* Amyloid Plaque-Associated Oxidative Degradation of Uniformly Radiolabeled Arachidonic Acid. *ACS Chem Neurosci* **7**, 367-377, doi:10.1021/acschemneuro.5b00316 (2016).

59 Picklo, M. J., Olson, S. J., Markesbery, W. R. & Montine, T. J. Expression and activities of aldo-keto oxidoreductases in Alzheimer disease. *J Neuropathol Exp Neurol* **60**, 686-695, doi:10.1093/jnen/60.7.686 (2001).

60 Chen, J. *et al.* Association Between Aldehyde dehydrogenase-2 Polymorphisms and Risk of Alzheimer's Disease and Parkinson's Disease: A Meta-Analysis Based on 5,315 Individuals. *Front Neurol* **10**, 290, doi:10.3389/fneur.2019.00290 (2019).

61 Greene, S. J., Killiany, R. J. & Alzheimer's Disease Neuroimaging, I. Subregions of the inferior parietal lobule are affected in the progression to Alzheimer's disease. *Neurobiol Aging* **31**, 1304-1311, doi:10.1016/j.neurobiolaging.2010.04.026 (2010).

62 Roquet, D. *et al.* Insular atrophy at the prodromal stage of dementia with Lewy bodies: a VBM DARTEL study. *Sci Rep* **7**, 9437, doi:10.1038/s41598-017-08667-7 (2017).

63 Guo, Y. J. *et al.* The ALDH2 Glu504Lys polymorphism is associated with coronary artery disease in Han Chinese: Relation with endothelial ADMA levels. *Atherosclerosis* **211**, 545-550, doi:10.1016/j.atherosclerosis.2010.03.030 (2010).

64 Ma, C. *et al.* Associations between aldehyde dehydrogenase 2 (ALDH2) rs671 genetic polymorphisms, lifestyles and hypertension risk in Chinese Han people. *Sci Rep* **7**, 11136, doi:10.1038/s41598-017-11071-w (2017).

65 Black, S., Gao, F. & Bilbao, J. Understanding white matter disease: imaging-pathological correlations in vascular cognitive impairment. *Stroke* **40**, S48-52, doi:10.1161/STROKEAHA.108.537704 (2009).

66 Tabor, H. K., Risch, N. J. & Myers, R. M. Candidate-gene approaches for studying complex genetic traits: practical considerations. *Nature Reviews Genetics* **3**, 391-397, doi:10.1038/nrg796 (2002).

# Acknowledgments

This work was supported by the National Research Foundation of Korea (NRF), funded by the Ministry of Science and ICT (NRF-2019R1A5A2026045), and a grant of the MD-Phd/Medical Scientist Training Program through the Korea Health Industry Development Institute (KHIDI), funded by the Ministry of Health & Welfare, Republic of Korea. This study was conducted with biospecimens and data from the biobank of the Chronic Cerebrovascular Disease Consortium in Korea and the consortium was also supported and funded by the Korea Centers for Disease Control and Prevention (#4845-303).

# Author contributions

Y.H.C., B.P. and S.J.S.: research conception and design. Y.H.C.: manuscript-writing of the first draft. H.L.: statistical analysis execution. B.P.: statistical analysis-review and critique. J.W.C. and N.R.K.: imaging analysis execution. C.H.H., S.W.S., S.H.C., E.J.K., B.C.K., S.Y.K. and J. C.: data acquisition and checking the quality of data. H.W.R., J.H.H., B.P. and S.J.S.: manuscript-review and critique. All authors read and approved the final manuscript.

# Competing interest

The authors declare no competing interests.

# Additional information

**Supplementary Information** The online version contains supplementary material available at

**Correspondence** and requests for materials should be addressed to B.P. or S.J.S.

**Table 1. Characteristics of the study participants grouped according to the SUVr of amyloid PET and the presence of rs671 A allele (N = 179)**

|  | **A(-)AC (N=25)** | **A(-)GG (N=62)** | ***p^a^*** |  | **A(+)AC (N=31)** | **A(+)GG (N=61)** | ***p^a^*** |
| --- | --- | --- | --- | --- | --- | --- | --- |
|  | **Mean(SD)/N(%)** | **Mean(SD)/N(%)** |  |  | **Mean(SD)/N(%)** | **Mean(SD)/N(%)** |  |
| **Age^d^ (year)** | 71.80 (7.35) | 70.77 (7.38) | 0.558 |  | 72.00 (8.03) | 74.508 (7.15) | 0.131 |
| **Sex** |  |  | 0.062 |  |  |  | 0.609 |
| Male (N, %) | 13 (52.0%) | 19 (30.6%) |  |  | 11 (35.5%) | 25 (41.0%) |  |
| Female (N, %) | 12 (48.0%) | 43 (69.4%) |  |  | 20 (64.5%) | 36 (59.0%) |  |
| **Education year** | 8.58 (5.29) | 8.68 (4.37) | 0.93 |  | 9.95 (5.45) | 9.05 (5.00) | 0.43 |
| **HTN (N, %)** | 15 (60.0%) | 36 (58.1%) | 0.868 |  | 18 (58.1%) | 33 (54.1%) | 0.718 |
| **Dyslipidemia (N, %)** | 8 (32.0%) | 31 (50.0%) | 0.127 |  | 10 (32.3%) | 20 (32.8%) | 0.959 |
| **DM (N, %)** | 8 (32.0%) | 11 (17.7%) | 0.145 |  | 3 (9.7%) | 11 (18.0%) | 0.292 |
| **FGCRS (%)** | 16.56 (4.30) | 14.19 (3.91) | 0.015 |  | 14.94 (4.84) | 15.03 (4.19) | 0.921 |
| **Lifetime alcohol drinking** |  |  | 0.201 |  |  |  | 0.004 |
| Non-drinker (N, %) | 20 (80.0%) | 41 (66.1%) |  |  | 27 (87.1%) | 36 (59.0%) |  |
| Drinker (N, %) | 5 (20.0%) | 21 (33.9%) |  |  | 4 (12.9%) | 25 (41.0%) |  |
| **APOE-ε4 allele carrier^e, f^ (N, %)** | 5 (20.0%) | 11 (17.7%) | 0.806 |  | 22 (71.0%) | 31 (50.8%) | 0.065 |
| **TIV (cc)** | 1.42 (0.14) | 1.41 (0.15) | 0.65 |  | 1.40 (0.14) | 1.38 (0.12) | 0.624 |
| **SUVr_Global^c, d^** | 0.58 (0.04) | 0.58 (0.03) | 0.733 |  | 0.87 (0.12) | 0.91 (0.14) | 0.107 |
| **WMH_peri (mL)** | 6.84 (8.50) | 8.54 (10.31) | 0.492 |  | 8.00 (11.76) | 9.48 (9.38) | 0.524 |
| **WMH_deep (mL)** | 2.82 (6.17) | 4.51 (7.58) | 0.354 |  | 4.23 (8.27) | 4.37 (10.32) | 0.949 |
| **Cognitive diagnosis^e^ (N, %)** |  |  | 0.228 |  |  |  | 0.124 |
| Alzheimer's disease | 3 (12.0%) | 2 (3.2%) |  |  | 18 (58.1%) | 32 (52.5%) |  |
| Vascular dementia | 1 (4.0%) | 10 (16.1%) |  |  | 2 (6.5%) | 1 (1.6%) |  |
| Dementia with Lewy bodies | 0 (0.0%) | 0 (0.0%) |  |  | 0 (0.0%) | 1 (1.6%) |  |
| MCI | 17 (68.0%) | 35 (56.5%) |  |  | 9 (29.0%) | 27 (44.3%) |  |
| Subjective memory impairment | 4 (16.0%) | 14 (22.6%) |  |  | 2 (6.5%) | 0 (0.0%) |  |
| Other^b^ | 0 (0.0%) | 1 (1.6%) |  |  | 0 (0.0%) | 0 (0.0%) |  |
| **K-MMSE (score)** | 25.12 (3.86) | 24.68 (4.10) | 0.65 |  | 21.61 (5.92) | 21.46 (5.49) | 0.902 |
| **CDR (score)** |  |  | 0.518 |  |  |  | 0.731 |
| 0 | 4 (16.0%) | 5 (8.5%) |  |  | 0 (0.0%) | 0 (0.0%) |  |
| 0.5 | 19 (76.0%) | 44 (74.6%) |  |  | 18 (58.1%) | 39 (65.0%) |  |
| 1 | 1 (4.0%) | 8 (13.6%) |  |  | 10 (32.3%) | 14 (23.3%) |  |
| 2 | 1 (4.0%) | 1 (1.7%) |  |  | 2 (6.5%) | 6 (10.0%) |  |
| 3 | 0 (0.0%) | 1 (1.7%) |  |  | 1 (3.2%) | 1 (1.7%) |  |
| **CDR Sum of Box^c, d^ (mean, SD)** | 1.74 (3.00) | 2.20 (2.52) | 0.477 |  | 4.40 (4.21) | 3.92 (3.48) | 0.558 |
| **rs671group (N, %)** |  |  | < 0.001 |  |  |  | < 0.001 |
| AA | 5 (20.0%) | 0 (0.0%) |  |  | 3 (9.7%) | 0 (0.0%) |  |
| AG | 20 (80.0%) | 0 (0.0%) |  |  | 28 (90.3%) | 0 (0.0%) |  |
| GG | 0 (0.0%) | 62 (100.0%) |  |  | 0 (0.0%) | 61 (100.0%) |  |

APOE, apolipoprotein E; HTN, hypertension; DM, diabetes mellitus; FGCRS, Framingham general cardiovascular risk score; TIV, total intracranial volume; SUVr, standardized uptake value ratio; WMH, white matter hyperintensities; MCI, mild cognitive impairment; K-MMSE, Korean-Mini Mental Status Examination; CDR, Clinical Dementia Rating

^a^ Student’s *t*-test was conducted for continuous variables. Chi-square tests were performed for categorical variables.

^b^ Unspecified sleep disorder

^c^ Variable means 1 < 3 as a result of the multiple comparison with Bonferroni correction, *p* < 0.05.

^d^ Variable means 2 < 4 as a result of the multiple comparison with Bonferroni correction, *p* < 0.05.

^e^ Variable means 1 vs. 3 as a result of the multiple comparison with Bonferroni correction, *p* < 0.05.

^f^ Variable means 2 vs. 4 as a result of the multiple comparison with Bonferroni correction, *p* < 0.05.

**Table 2. Cognitive function of study participants grouped according to the SUVr of amyloid PET and the presence of rs671 A allele (N = 179)**

|  | **A(-) (SUVr < 0.65)** | | **A(+) (SUVr > 0.65)** | | ***P^a^*** | **Post-hoc** |
| --- | --- | --- | --- | --- | --- | --- |
| **Cognitive function (Z-score)** | **AC (N=25)** | **GG (N=62)** | **AC (N=31)** | **GG (N=61)** |  |  |
|  | **Mean (SD)** | **Mean (SD)** | **Mean (SD)** | **Mean (SD)** |  |  |
| Attention function | -0.454 (0.931) | -0.070 (0.906) | -0.526 (1.026) | -0.499 (1.129) | 0.073 |  |
| Language function | -0.616 (1.891) | -0.498 (1.416) | -1.228 (2.286) | -1.137 (2.055) | 0.188 |  |
| Visuospatial function | -1.114 (1.905) | -0.665 (1.472) | -1.856 (3.380) | -2.224 (2.663) | 0.003 | 2>4 |
| Memory function | -0.952 (1.459) | -1.054 (1.196) | -2.292 (1.459) | -2.222 (1.573) | <0.001 | 1>3, 2>4 |
| Frontal/executive function | -1.569 (2.004) | -0.875 (1.467) | -2.166 (2.359) | -2.515 (2.183) | <0.001 | 2>4 |

AC, A-carrier; PET, positron emission tomography SD, standard deviation; SUVr, standardized uptake value ratio.

All values are z-score results.

^a^ ANOVA test was conducted for continuous variables.

**Table 3. Results of regressions analyses of the interaction effect of amyloid deposition and rs671 polymorphism on brain cortical thickness, only showing significant regions**

| **Regions** | **Estimate^a^** | **Standard  error^a^** | **Unadjusted  *P*** | **FDR corrected  *P*** |
| --- | --- | --- | --- | --- |
| Lt. cuneus | -0.143 | 0.049 | 0.004 | 0.043 |
| Lt. pars opercularis | -0.162 | 0.047 | 0.001 | 0.038 |
| Lt. pars triangularis | -0.134 | 0.045 | 0.004 | 0.043 |
| Lt. rostral middle frontal | -0.162 | 0.049 | 0.001 | 0.038 |
| Lt. superior parietal | -0.145 | 0.045 | 0.002 | 0.038 |
| Lt. insula | -0.186 | 0.066 | 0.005 | 0.047 |
| Rt. inferior parietal | -0.152 | 0.055 | 0.006 | 0.048 |
| Rt. pars triangularis | -0.163 | 0.055 | 0.004 | 0.043 |
| Rt. superior parietal | -0.139 | 0.048 | 0.005 | 0.045 |

FDR, false discovery rate; APOE, apolipoprotein E; CDR, clinical dementia rating.
Adjusted for age, sex, education year, total intracranial volume, APOE-ε4 carrier status, lifetime drinking status, Framingham general cardiovascular risk score, CDR sum of box, white matter hyperintensity score of peripheral and deep white matter.
^a^ Values from amyloid deposition and rs671 polymorphism interaction term in multiple regression model.

**Table 4. Lsmeans of cortical thickness in the four groups and among-group comparisons in each brain region**

| Regions | Lsmeans of cortical thickness(SE) | | | |  | | | |
| --- | --- | --- | --- | --- | --- | --- | --- | --- |
|  | A (-) | | A (+) | | *p* (Bonferroni) | | | |
|  | AC | GG | AC | GG | 1 vs 2 | 3 vs 4 | 1 vs 3 | 2 vs 4 |
| **Lt. cuneus** | 1.748(0.034) | 1.856(0.022) | 1.892(0.028) | 1.857(0.019) | 0.016**^*^** | 1 | 0.005**^*^** | 1 |
| **Lt. pars opercularis** | 2.336(0.033) | 2.468(0.021) | 2.460(0.027) | 2.431(0.018) | 0.001**^*^** | 1 | 0.015**^*^** | 0.726 |
| **Lt. pars triangularis** | 2.237(0.031) | 2.359(0.020) | 2.359(0.026) | 2.348(0.017) | 0.002**^*^** | 1 | 0.013**^*^** | 1 |
| **Lt. rostral middle frontal** | 2.205(0.034) | 2.325(0.022) | 2.310(0.028) | 2.269(0.019) | 0.006**^*^** | 0.837 | 0.073 | 0.227 |
| **Lt. superior parietal** | 2.049(0.031) | 2.175(0.020) | 2.104(0.026) | 2.086(0.017) | 0.001**^*^** | 1 | 0.697 | 0.005**^*^** |
| **Lt. insula** | 2.652(0.046) | 2.781(0.030) | 2.830(0.038) | 2.774(0.025) | 0.044**^*^** | 0.807 | 0.013**^*^** | 1 |
| **Rt. Inferior parietal** | 2.298(0.038) | 2.411(0.025) | 2.348(0.032) | 2.308(0.021) | 0.032**^*^** | 1 | 1 | 0.010**^*^** |
| **Rt. pars triangularis** | 2.254(0.038) | 2.375(0.025) | 2.379(0.032) | 2.338(0.021) | 0.018**^*^** | 1 | 0.052 | 1 |
| **Rt. superior parietal** | 2.062(0.033) | 2.169(0.022) | 2.112(0.028) | 2.080(0.019) | 0.016**^*^** | 1 | 1 | 0.010**^*^** |

Lsmeans, Least-Squares means.

SE, Standard errors.

We marked * for p-value below 0.05 after Bonferroni correction.

**Figure Legends**

**Figure 1.**

The results of the regression analyses of the interaction effect of amyloid deposition and rs671 polymorphism on brain cortical thickness using the Desikan-Killiany atlas and violin plot. After correction at FDR < 0.05, nine regions were observed, with six and three in the left and right hemispheres, respectively (Lt. cuneus, Lt. pars opercularis, Lt. pars triangularis, Lt. rostral middle frontal, Lt. superior parietal, Lt. insula, Rt. Inferior parietal, Rt. pars triangularis, Rt. superior parietal). The nine regions in the brain maps were color-coded using scales of -log10 (uncorrected p) from the multiple regression analysis. The violin plot was made with the estimated values obtained from regression analysis, and Lsmeans with standard errors were marked with error bars. Brain maps and violin plot was visualized using R-packages ggseg (https://github.com/ggseg/ggseg)^41^ and ggplot2^42^.


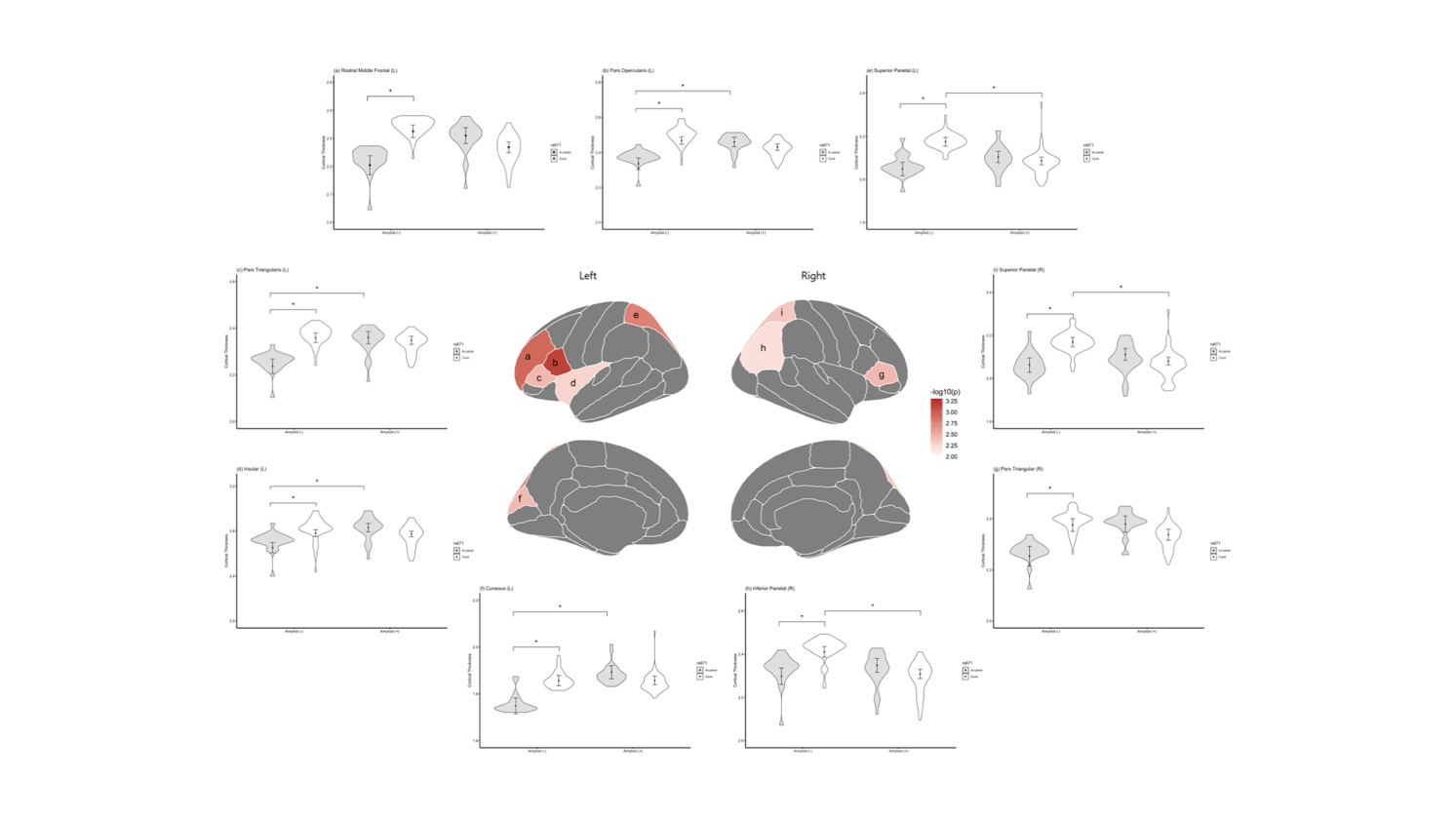

Supplement: Supplementary file 1 — Supplementary Tables. [file 41598_2021_98834_MOESM1_ESM.docx]
